# A comprehensive review of deep brain stimulation for drug-resistant epilepsy

**DOI:** 10.3389/fneur.2026.1884342

**Published:** 2026-07-28

**Authors:** Ryota Sasaki, Masako Kinoshita, Abbas F. Sadikot, Ichiro Nakagawa

**Affiliations:** 1 Department of Neurosurgery, Nara Medical University, Kashihara, Japan; 2 Department of Neurology & Neurosurgery, Montreal Neurological Institute, McGill University, Montreal, QC, Canada; 3 Department of Neurology, National Hospital Organization Utano National Hospital, Kyoto, Japan

**Keywords:** deep brain stimuation, drug resistant epilepsy (DRE), epilepsy, etiology, neuromodulation, palliative surgery, status epilepticus (SE), thalamus

## Abstract

**Background:**

Deep brain stimulation (DBS) has emerged as a palliative neurosurgical treatment option for drug-resistant epilepsy (DRE), particularly in patients who are not candidates for resective surgery or who continue to experience seizures after surgical intervention. Multiple thalamic and extrathalamic targets have been investigated; however, optimal target selection remains challenging and requires a comprehensive understanding of epileptogenic networks.

**Objective:**

To provide a comprehensive overview of current evidence on DBS for DRE and refractory status epilepticus, with a focus on anatomical targets, mechanisms of action, clinical outcomes, and considerations for target selection.

**Methods:**

We reviewed the existing literature on DBS in epilepsy, including randomized controlled trials, observational studies, meta-analyses, and relevant experimental data. Major targets—including the anterior nucleus of the thalamus (ANT), centromedian nucleus (CM), pulvinar, mediodorsal nucleus (DM), subthalamic nucleus (STN), and other emerging targets—were examined in terms of connectivity, proposed mechanisms, and clinical efficacy.

**Results:**

Among available targets, ANT-DBS has the strongest clinical evidence, demonstrating sustained seizure reduction in randomized trials and long-term follow-up studies. CM-DBS shows particular promise in generalized epilepsy, especially Lennox–Gastaut syndrome, likely through modulation of thalamocortical and reticular networks. The pulvinar has emerged as a potential target for temporal and posterior quadrant epilepsies, reflecting its extensive cortical connectivity. Other targets, including the DM, STN, hippocampus, hypothalamus, nucleus accumbens, and cerebellum, have shown variable efficacy in smaller studies and may be relevant for specific epilepsy subtypes or network configurations. Across targets, therapeutic effects are likely mediated by modulation of distributed epileptogenic networks involving limbic, sensorimotor, and arousal systems.

**Conclusion:**

DBS represents an important therapeutic option for DRE, expanding the scope of neuromodulation beyond traditional surgical approaches. Optimal outcomes depend on individualized target selection based on seizure semiology, network characteristics, and anatomical considerations. As clinical experience and technological advances continue to evolve, further studies are required to refine patient selection, improve targeting strategies, and optimize stimulation paradigms.

## 1 Introduction

Epilepsy is a chronic neurological disorder, with a reported prevalence of approximately 4 to 8 per 1,000 individuals in the general population (1). Seizures can be controlled with anti-seizure medications (ASMs) in approximately 70% of patients; however, the remaining 30% are considered to have drug-resistant epilepsy (DRE), for which epilepsy surgery should be considered (2). Epilepsy surgery can lead to seizure reduction or remission and thereby improve quality of life. When a resectable epileptogenic focus is identified, focal resection is selected as a potentially curative surgical treatment. In contrast, when a patient has the multiple foci, the widespread epileptogenic network, or the focus involving eloquent areas whose resection would result in substantial functional loss, palliative surgical treatment is considered. In recent years, deep brain stimulation (DBS) has emerged as one of the treatment options in this palliative setting. DBS was first applied for tremor in 1980 and subsequently evolved as an established treatment for movement disorders, including Parkinson’s disease and dystonia (3, 4). DBS for epilepsy has been investigated for more than five decades, culminating in approval by the U.S. Food and Drug Administration (FDA) in 2018 (5–7). Whereas DBS for movement disorders is generally delivered as continuous stimulation, DBS for epilepsy is more commonly administered using cyclic stimulation paradigms. DBS is a therapeutic intervention that modulates neural activity through stimulation of specific deep brain nuclei and, together with vagus nerve stimulation (VNS) and responsive neurostimulation (RNS), is categorized as a form of neuromodulation therapy. Although the therapeutic mechanism of DBS remains unclear, the most widely accepted hypothesis is that its effects arise from modulation of abnormal neural network activity (8, 9). This concept is consistent with the view that many disorders treated with DBS are fundamentally disorders of neural networks.

**Figure 1:**
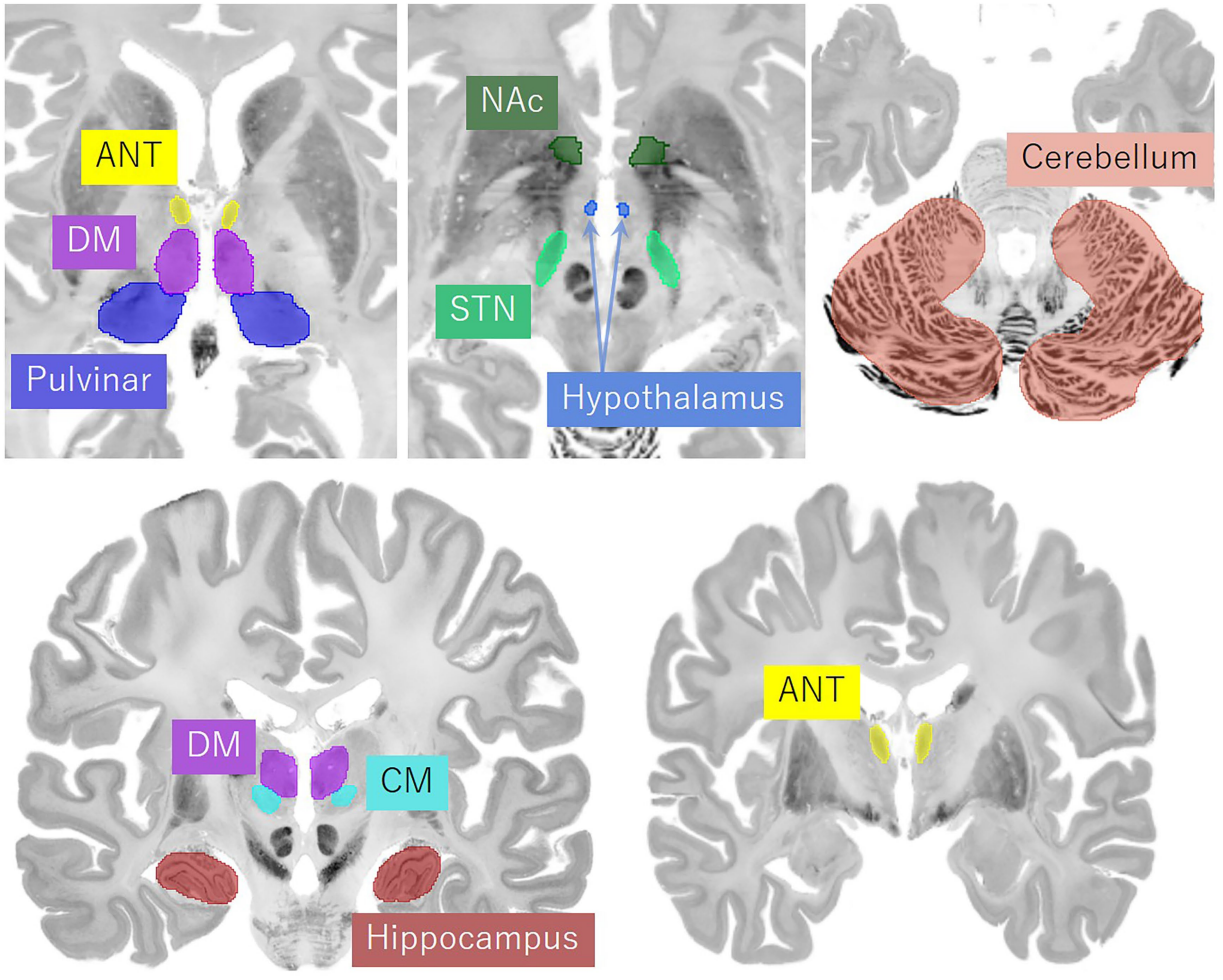
Targets investigated for DBS in epilepsy. The upper row shows axial views, and the lower row shows coronal views. Brain images were derived from 200-nm sections from the BigBrain Project (137). Each target was reconstructed using 3D Slicer. The brain images were normalized to MNI152NLin2009bAsym space using the advanced normalization tools method, and segmentation was performed using both manual delineation and nnU-Net with reference to histological findings (138, 139). DBS: deep brain stimulation, ANT: anterior nucleus of the thalamus, CM: centromedian nucleus, DM: mediodorsal nucleus, NAc: nucleus accumbens, STN: subthalamic nucleus.

Various anatomical targets have been proposed for DBS in the treatment of epilepsy, and the optimal target should be selected according to the clinical characteristics of each case (Figure 1). At present, the most widely performed target for epilepsy is the anterior nucleus of the thalamus (ANT). In stimulation of the ANT, it has been reported that seizure suppression is greater in patients with stronger functional connectivity between the thalamus and the seizure onset zone, highlighting the importance of rational target selection tailored to the seizure network of each patient (10, 11). In addition, DBS may confer secondary benefits including improvements in attention and depressive symptoms. However, device- or stimulation-related adverse events potentially lead to social withdrawal and isolation (12, 13). Therefore, it is essential to understand the anatomical and physiological basis of side effects associated with stimulation and to make every effort to prevent them whenever possible.

**Figure 2:**
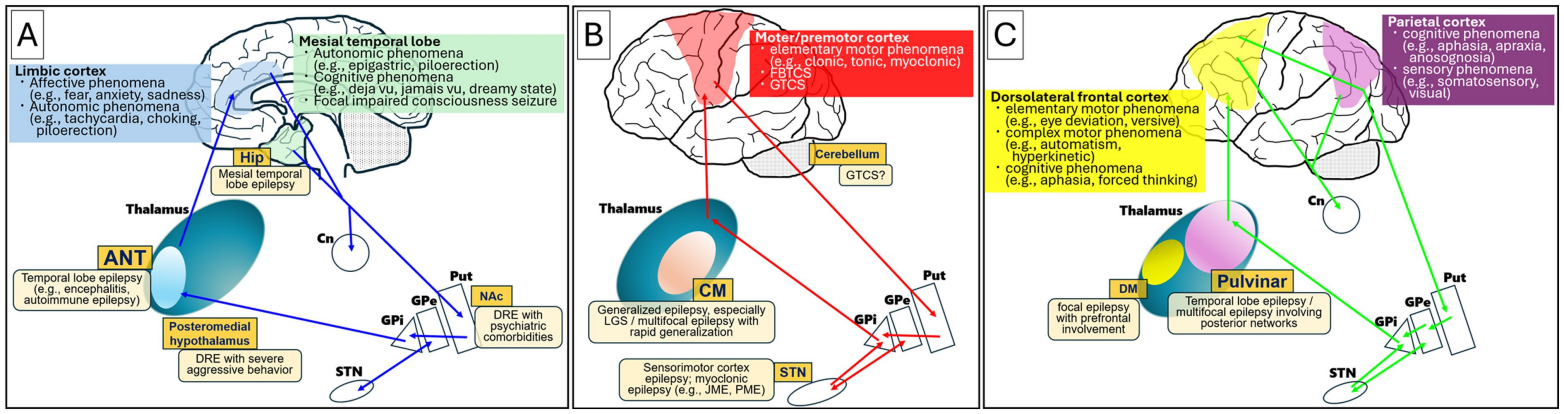
Relationship between seizure semiologies and the cortico–basal ganglia–thalamic pathways **(A)** Limbic circuit, **(B)** Motor circuit, and **(C)** Associative circuit (140–145). The cortical regions involved in each circuit are shaded on the corresponding brain schematics. The names of the cortical regions are shown in bold within the adjacent boxes, together with the associated seizure semiologies. In the orange squares, bold text indicates the principal DBS targets, with the etiologies for which each target is considered effective shown adjacent to them. Selection of DBS targets associated with the relevant neural network may enable more effective treatment. DBS: Deep brain stimulation; ANT: Anterior nucleus of the thalamus; CM: Centromedian nucleus; Cn: Caudate nucleus; DM: Mediodorsal nucleus; DRE: Drug-resistant epilepsy; FBTCS: focal to bilateral tonic-clonic seizure; GPe: Globus pallidus externa; GPi: Globus pallidus interna; GTCS: Generalized tonic–clonic seizure; Hip: Hippocampus; JME: Juvenile myoclonic epilepsy; LGS: Lennox–Gastaut syndrome; NAc: Nucleus accumbens; PME: Progressive myoclonic epilepsy; Put: Putamen; STN: Subthalamic nucleus.

Although numerous reviews on DBS for epilepsy have been published, none have specifically addressed DBS target selection in relation to seizure semiology (14–30). We hypothesized that considering the neural networks underlying seizure semiology may facilitate a more rational selection of DBS targets (Figure 2). In this review, we comprehensively examine the current evidence regarding DBS for DRE, with the aim of facilitating optimal target selection in clinical practice (Table 1).

**Table 1:** Summary of DBS targets for drug-resistant epilepsy.

Target	Seizure reduction rate	Effective etiology/seizure type	Adverse events/complications	References
ANT	~40% at 3 months; ~56% at 2 years; up to ~75% at 7 years	Temporal lobe epilepsy, especially mesial temporal and limbic network-related epilepsy; post-encephalitic or autoimmune epilepsy	Depression, memory impairment, anxiety; stimulation-related discomfort (pain, paresthesia); psychiatric symptoms (often manageable with parameter adjustment)	Salanova et al. (13) and Fisher et al. (40)
CM	~46.7% at 6 months; ~69%	Generalized epilepsy, especially LGS; multifocal epilepsy with rapid generalization	Transient somnolence (common, improves with continued stimulation); generally well tolerated	Dhaliwal et al. (54) and Dalic et al. (76)
Pulvinar	~50–90%	Temporal lobe epilepsy; PQE; multifocal epilepsy involving posterior networks	Mild cognitive effects (auditory attention, working memory), insomnia; generally non-disabling	Pizzo et al. (88) and Chandran et al. (89)
DM	Limited clinical data; variable	Epilepsy involving frontolimbic networks; potential role in focal epilepsy with prefrontal involvement	Cognitive effects (e.g., working memory impairment with stimulation); insufficient data	Yang et al. (107) and Peräkylä et al. (108)
STN	~33–100%; ~64% average in focal epilepsy	Sensorimotor cortex epilepsy; myoclonic epilepsy (e.g., JME, PME); possibly absence epilepsy (theoretical)	Dyskinesia, dizziness; stimulation-related motor side effects (dose-dependent)	Cui et al. (111) and Wille et al. (113)
Hip	~66% (FAS), ~91% (FIAS); seizure freedom up to ~32%	Mesial temporal lobe epilepsy, especially when resection is not feasible (e.g., dominant hemisphere)	Generally well tolerated; no significant cognitive decline reported (possible mild cognitive improvement)	Cukiert et al. (120) and Cukiert et al. (121)
Posteromedial hypothalamus	~89.6%	DRE with severe aggressive behavior; limbic network-related epilepsy	Limited data; potential autonomic and behavioral effects	Benedetti-Isaac et al. (123)
Nucleus accumbens	~37.5%	DRE with psychiatric comorbidities; temporal lobe epilepsy (network modulation)	Limited data; potential mood and motivational effects	Schmitt et al. (125)
Cerebellum	Inconsistent; unclear efficacy	Generalized tonic–clonic seizures (historically studied)	Not well established; inconsistent outcomes	Fountas et al. (127)

DBS: deep brain stimulation, ANT: anterior nucleus of the thalamus, CM: centromedian nucleus, DM: mediodorsal nucleus, STN: subthalamic nucleus, Hip: hippocampus, FAS: focal aware seizure, FIAS: focal impaired awareness seizure, LGS: Lennox–Gastaut syndrome, PQE: posterior quadrant epilepsy, JME: juvenile myoclonic epilepsy, PME: progressive myoclonic epilepsy, DRE: drug-resistant epilepsy.

## 2 Overview of DBS for epilepsy

### 2.1 Target selection strategies

Historically, epilepsy surgery was considered to be achieved by resecting the abnormally hyperexcitable cortical region, the so-called epileptogenic zone (31). In recent years, however, surgical treatment has increasingly shifted toward addressing epileptogenic networks that may extend beyond adjacent cortical areas (32). These epileptogenic networks comprise complex circuits, including the basal ganglia, but they can be identified by carefully integrating each patient’s seizure semiology with anatomical and physiological knowledge. Because DBS exerts its therapeutic effects by modulating abnormal neural networks, a deep understanding of seizure semiology is essential to achieve optimal outcomes. In 2025, the ILAE revised its classification of epileptic seizures to improve clinical applicability and diagnostic accuracy (33). In this updated classification, the temporal evolution of seizure semiology is described as part of the seizure description, thereby further emphasizing the importance of epileptogenic networks. Accordingly, target selection for DBS in epilepsy should ideally be guided not only by epilepsy type or syndrome, but also by identification of the neural networks involved, based on seizure semiology.

### 2.2 Adaptive DBS

Adaptive DBS (aDBS) enables real-time adjustment of stimulation parameters based on local field potential (LFP) recorded from intracranial leads. LFP is recorded using bipolar sensing contacts positioned to flank one or two monopolar stimulation contacts. Because these LFPs contain a mixture of signals across multiple frequency bands, frequency-domain analysis can be performed using fast Fourier transform. In addition, patients or caregivers can mark events of interest using the patient programmer, and the corresponding event timestamps are stored in the device. At present, aDBS has been actively implemented in clinical practice for subthalamic nucleus (STN)-DBS in Parkinson’s disease, using protocols in which stimulation parameters are modulated according to changes in beta-band power recorded from the STN (34). In epilepsy, the application of aDBS has so far been largely limited to chronic sensing for the detection of biomarkers such as seizures and interictal epileptiform discharges (35). This functionality may enable accurate tracking of seizure events. However, this approach has several limitations: contacts used for sensing cannot be used for stimulation, thereby narrowing the available options for stimulation programming, and the configuration of adaptive DBS itself can be time-consuming. In parallel, thalamic electrophysiological recordings using SEEG have been increasingly performed, providing new insights into ictal and interictal electrophysiological dynamics (36–39). The continued accumulation of these biomarkers may, in the future, enable aDBS to optimize stimulation for individual patients, detect seizures, and monitor the excitability of epileptogenic networks.

### 2.3 Programming considerations

DBS programming involves numerous parameters, including active contacts and stimulation amplitude, as well as pulse width, frequency, and whether stimulation is delivered continuously or intermittently, resulting in a virtually unlimited number of possible combinations. The commonly used ANT-DBS settings of 90 μs, 145 Hz, and 1 min ON/5 min OFF are derived from the standard protocol whose efficacy was demonstrated in the SANTE trial; however, no comparative trial has directly shown that each of these individual parameters is optimal (40). A meta-analysis of DBS stimulation parameters reported that the proportion of patients achieving a ≥ 50% reduction in seizure frequency was higher with a stimulation frequency of 130 Hz (75%), continuous stimulation (75%), and voltage or current settings of ≤5 (73%), although the underlying reasons remain unclear (41). Low-frequency stimulation has also been considered to carry a risk of seizure worsening; however, Alcala-Zermeno et al. demonstrated efficacy and safety comparable to those of the SANTE stimulation protocol in 16 patients treated with continuous stimulation at 7 Hz and a pulse width of 200 μs (42). Thus, the optimal stimulation settings have not yet been established, and it is important to adjust stimulation parameters for each patient and adopt an individualized therapeutic approach. Although many centers initiate stimulation early after surgery, stimulation can be started at the target intensity from the outset if no adverse events occur. In some cases, however, a microlesion effect caused by the physical insertion of the electrode (40, 43), independent of electrical stimulation, may result in transient seizure improvement. Because this effect lasts for up to three postoperative months, it should be carefully considered when adjusting stimulation parameters.

### 2.4 Comparisons with other neuromodulation modalities such as RNS and VNS

Other neuromodulation therapies for drug-resistant epilepsy include vagus nerve stimulation (VNS) and responsive neurostimulation (RNS). Since its approval by the U.S. Food and Drug Administration (FDA) in 1997, VNS has been widely used worldwide (16, 17, 23). Stimulation of the vagus nerve activates afferent sensory fibers projecting to the nucleus tractus solitarius, which in turn projects to structures including the locus coeruleus, raphe nuclei, limbic and forebrain regions, somatosensory cortex, hypothalamus, and thalamic nuclei (44). Through these pathways, VNS is thought to exert antiseizure effects by increasing noradrenergic and serotonergic activity. Because VNS is technically straightforward and minimally invasive, it can be applied across a wide age range and even in patients with poor general condition. In addition, VNS has demonstrated efficacy for depression and may therefore benefit patients suffering from both epilepsy and depression (45). RNS was developed as a closed-loop system that detects seizure-related electrographic activity and delivers electrical stimulation in response. This therapy evolved from observations that brief pulses of electrical stimulation can terminate afterdischarges during cortical stimulation (46–49). In patients with no more than two seizure foci, one or two four-contact subdural cortical strip or depth electrodes are implanted near the seizure onset zone. The pulse generator is implanted within the skull, and clinicians program the device to detect and treat seizures. The pulse generator continuously analyzes electrocorticographic activity and, when ictal activity is detected, delivers electrical stimulation intended to abort seizure activity. RNS offers several advantages, including applicability to seizure foci located in eloquent or otherwise unresectable regions and the ability to collect long-term electrophysiological data.

No randomized controlled trial has directly compared DBS, VNS, and RNS. In a single-center retrospective study comparing ANT-DBS (*n* = 38), CM-DBS (*n* = 19), RNS (*n* = 30), and VNS (*n* = 40), the median follow-up duration was 26 months (IQR, 13–52), and the median seizure reduction rates were 63% for CM-DBS, 52% for ANT-DBS, 50% for RNS, and 50% for VNS (50). In focal epilepsy, a meta-analysis including 642 patients found that the 1-year seizure reduction rates were significantly greater with RNS (66.3%) and DBS (58.4%) than with VNS (32.9%; *p* < 0.01), although this difference diminished over longer-term follow-up (51). For generalized epilepsy, a meta-analysis of 20 studies including 510 patients treated with VNS and 87 treated with DBS showed seizure reductions of 64.8% with DBS over a mean follow-up of 23.1 months and 48.3% with VNS over a mean follow-up of 39.1 months; this difference was statistically significant (*p* = 0.02) (52). RNS was not included in this analysis because of insufficient data, although the available results were considered promising. The choice among these devices depends on multiple factors, including the feasibility of intracranial electroencephalographic evaluation, the identification and location of the seizure-onset zone, the number of seizure foci, comorbidities, and patient preferences.

## 3 Characteristics of each DBS target

### 3.1 Anterior nucleus of the thalamus (ANT)

In the first report of the Stimulation of the Anterior Nucleus of the Thalamus for Epilepsy (SANTE) trial in 2010 involving 110 patients with DRE, participants with device implantation were randomized to stimulation or control groups after a 3-month baseline period. At 3 months of the stimulation period, the median seizure reduction was 40.4% in the stimulation group compared to 14.5% in the control group, demonstrating a significant therapeutic effect. In the stimulation group, the median seizure reduction improved to 56% at 2 years (40). Based on these results, ANT-DBS was approved in the United States in 2018 for patients aged ≥18 years with DRE and was subsequently covered by insurance in Japan in December 2023 for focal DRE. Long-term follow-up data from the SANTÉ trial showed a median seizure reduction of 75% at 7 years, suggesting that efficacy may increase with longer stimulation duration (13). In post-marketing data from the Medtronic Registry for Epilepsy (MORE), the median seizure reduction was 33.1% at 2 years and 55.1% at 5 years (53). The relatively lower efficacy observed in MORE compared with SANTÉ may be attributable to differences in patient populations, including a lower proportion of temporal lobe epilepsy (TLE) and a higher prevalence of multifocal epilepsy. According to the most recent systematic review, the mean seizure reduction rate with ANT-DBS was 64.28% over an average follow-up period of 52 months (range, 12–168 months) (54). The responder rate for ANT-DBS was reported to be 44.05% in patients followed for less than 2 years, compared with 67.63% in those with follow-up durations exceeding 2 years (54).

In addition to seizure reduction, ANT-DBS has been associated with significant improvements in attention, executive function, depression, tension/anxiety, overall mood disturbance, and delayed verbal memory (13, 55). These effects are thought to be related to activation of frontolimbic circuits. Such findings are consistent with reports of postoperative cognitive improvement associated with increased glucose metabolism in the dorsolateral prefrontal cortex and dorsomedial/ventromedial prefrontal cortices following subtemporal amygdalohippocampectomy for mesial TLE (56).

Regarding adverse events, Salanova et al. reported that, over a 5-year follow-up period, 37.3% of patients experienced depression, 27.3% reported memory impairment, and 11.8% had suicidal ideation (57). Notably, 66% of patients with depression had a prior history of depression, and 50% of those with memory impairment had pre-existing cognitive complaints. These findings suggest that ANT-DBS should be applied with caution in patients with a history of depression. Järvenpää et al. reported that stimulation-related acute depressive symptoms improved with reduction of stimulation voltage and adjustment of the active contacts (58). Thus, stimulation-induced psychiatric symptoms can generally be managed by modifying stimulation parameters.

As part of the Papez circuit, the ANT is connected to frontal and mesial frontotemporal regions (59). The ANT is subdivided into three subnuclei: the anterodorsal (AD), anteroventral (AV), and anteromedial (AM) nuclei (60). The AV nucleus has strong connections with the subiculum and retrosplenial cortex, whereas the AM nucleus is widely connected with the anterior cingulate cortex and orbitomedial prefrontal cortex; these connections are considered important for DBS targeting. A meta-analysis investigating the optimal stimulation site (“sweet spot”) within the ANT demonstrated that stimulation of the AV region adjacent to the mammillothalamic tract (MTT) was associated with the greatest seizure reduction, with the responsive stimulation site located approximately 1.6 mm anterior to that of non-responders (61). In preoperative planning for ANT-DBS, direct visualization of the ANT on conventional MRI remains challenging. Although attempts have been made to improve ANT visualization using quantitative susceptibility mapping (QSM), targeting strategies based on magnetization-prepared gradient echo (MPRAGE) and fast gray matter acquisition T1 inversion recovery (FGATIR) imaging are currently more widely adopted (62–64). These sequences enable visualization of surrounding white matter landmarks that delineate the ANT, including the external medullary lamina, internal medullary lamina, and MTT.

ANT-DBS is considered particularly effective for TLE, which is supported by the anatomical relationship that both the ANT and mesial temporal structures are components of the limbic system (40). Although the precise mechanisms underlying the antiseizure effects of ANT stimulation remain unclear, they are thought to involve multiple factors. In animal studies, ANT stimulation has been shown to reduce inflammation-related genes and cytokines, thereby preventing inflammation and apoptosis, suggesting the involvement of immunological mechanisms (65, 66). This is consistent with a 1-year follow-up study in 22 patients with DRE, which evaluated plasma levels of the pro-inflammatory cytokine interleukin-6 (IL-6) and the anti-inflammatory, neuroprotective cytokine interleukin-10 (IL-10). In this study, the preoperative IL-6/IL-10 ratio was higher in responders than in non-responders, and this ratio significantly decreased over time following DBS in the overall cohort (67). In addition, ANT stimulation has been reported to increase adenosine levels and inhibit the progression of kindling, suggesting a role for neurotransmitter modulation (68). Furthermore, FDG-microPET studies in rats receiving bilateral ANT stimulation demonstrated reversible increases in glucose uptake in the thalamus and hippocampus, along with decreased uptake in the cingulate and frontal cortices, indicating that alterations in cerebral metabolism may also contribute to its therapeutic effects (69). Recent studies have further shown that longitudinal analysis of local field potentials (LFPs) in patients receiving ANT-DBS for 4 years demonstrated progressive spectral changes in responders, characterized by reductions in low-frequency activity such as delta and theta bands, together with increases in higher-frequency activity including beta and gamma bands over time (70). These findings suggest that time-dependent neuroplastic mechanisms may be induced by ANT-DBS through multiple interacting factors.

Clinically, bilateral ANT stimulation is generally performed; however, unilateral stimulation may be effective in cases with a unilateral seizure focus. In a rat model with unilateral amygdala kindling, high-frequency stimulation of the ipsilateral ANT significantly reduced seizure frequency and duration, whereas stimulation of the contralateral ANT did not produce significant seizure suppression (71). In contrast, in a rat model of secondary generalized seizures induced by intraperitoneal pilocarpine injection, unilateral ANT stimulation failed to suppress seizures, whereas bilateral stimulation or lesioning of the ANT resulted in significant seizure reduction (72). There are efferent fibers from the ANT which project bilaterally, however, unilateral stimulation is generally not considered to be sufficient to achieve therapeutic effects on the contralateral side (73).

Taken together, these findings suggest that the most suitable candidates for ANT-DBS are patients with focal epilepsy involving bilateral mesial temporal structures, such as those following encephalitis or meningitis. Furthermore, as a recent report has demonstrated efficacy even in bilateral TLE following new-onset refractory status epilepticus, DRE secondary to autoimmune-mediated disease should also be considered a potential indication for ANT-DBS (74).

### 3.2 Centromedian nucleus (CM)

Following ANT, CM is the DBS target for which evidence is increasingly accumulating. A meta-analysis of 135 patients with DRE who underwent CM-DBS reported a mean seizure reduction of 69% over an average follow-up period of 65.25 months (range: 12–132 months) (54). CM-DBS is considered particularly promising for generalized epilepsy. Velasco et al. reported an open-label study of 13 patients with Lennox–Gastaut syndrome (LGS) treated with CM-DBS, demonstrating approximately 80% seizure reduction (75). In the ESTEL trial, a prospective double-blind randomized study of CM-DBS in LGS, the median seizure reduction at 6 months was 46.7% (range: 28–67%) (76). A characteristic adverse effect was transient postoperative somnolence, observed in approximately 60% of patients; however, with ongoing stimulation, 95% of these patients showed improvement in alertness. In a case series of five patients with drug-resistant idiopathic generalized epilepsy, CM-DBS resulted in seizure reduction of 60–100% in four patients in whom appropriate CM stimulation was achieved (77). In addition, RNS targeting similar networks has demonstrated relatively favorable seizure reduction rates in multifocal epilepsy and generalized epilepsy with early evolution to focal to bilateral tonic–clonic seizures (78, 79). The CM is an intralaminar nucleus located along the lateral wall of the third ventricle (80). Functional connectivity analyses based on fMRI have demonstrated connections with the anterior cingulate cortex, prefrontal cortex, precentral gyrus, postcentral gyrus, insular cortex, mesial temporal lobe, and occipital lobe (24). In the ESTEL trial, EEG-fMRI performed in young adults with LGS identified significant connectivity between the CM and the sensorimotor cortex, premotor cortex, basal ganglia, brainstem, cerebellum, and limbic system (81). This EEG-fMRI analysis further revealed that generalized paroxysmal fast activity in LGS originates in the cerebral cortex, propagates to the brainstem via corticoreticular pathways, and subsequently involves the CM with a temporal delay (82).

Because the CM is small and lacks clear anatomical boundaries, precise targeting can be challenging. Sisterson et al. (83) employed an indirect targeting approach, positioning the target 1 mm anterior, 10 mm lateral, and 1 mm superior to the midpoint of the anterior commissure–posterior commissure line. Several MRI sequences have been explored to improve CM visualization, including magnetization-prepared 2 rapid acquisition gradient echo (MP2RAGE), QSM, and boundary enhancement gradient echo (EDGE) (81, 84, 85). Intraoperative microelectrode recordings suggest that the CM tends to exhibit lower background noise and spike firing rates compared with the ventrolateral nucleus, which may serve as an auxiliary indicator (81). However, this pattern is absent in approximately 20–25% of cases, and therefore should be interpreted with caution. An analysis of the “sweet spot” in patients with LGS has suggested that stimulation of the anteroinferolateral border (parvocellular region) of the CM is associated with marked seizure reduction (86). This finding is consistent with tractography studies indicating that favorable outcomes are associated with modulation of pathways related to the ascending reticular activating system including descending projections to the brainstem and cerebellum, as well as connections to the supplementary motor area and sensorimotor cortex (87).

### 3.3 Pulvinar

Although clinical experience with pulvinar-DBS remains limited, its efficacy is gradually being discussed. Pizzo et al. reported an interim analysis of the PULSE trial, an open-label prospective study of pulvinar-DBS in drug-resistant epilepsy, in which six patients showed a mean seizure reduction of 52% and a 50% responder rate of 40% (88). In a single-center study reported by Chandran et al., five patients with drug-resistant TLE or posterior quadrant epilepsy (PQE) achieved seizure reductions exceeding 50 to 90% following pulvinar-DBS (89). Stimulation-related adverse effects included mild impairments in auditory attention, working memory, and insomnia; however, these did not significantly interfere with daily functioning. It should be noted that a substantial proportion of patients in these studies had previously undergone other surgical interventions, such as resective surgery, VNS, or ANT-DBS. In a case report of multifocal epilepsy involving bilateral hippocampi and the right insula, pulvinar-DBS resulted in a 60% reduction in seizure frequency and enabled reliable seizure detection based on LFP analysis (90). Several case reports and small case series also described the use of pulvinar-targeted RNS, particularly in drug-resistant multifocal PQE, with reported seizure reductions ranging from approximately 50 to 100% (91–93).

The pulvinar is the largest subnucleus of the thalamus, accounting for approximately 40% of its volume, and exhibits extensive cortical connectivity. Animal studies have demonstrated that the lateral pulvinar is connected to the dorsolateral prefrontal cortex, inferior parietal cortex, and ventral visual stream areas, whereas the medial pulvinar is connected to the superior temporal cortex, insular cortex, cingulate cortex, and amygdala (94). Given its broad connections with the temporal, parietal, and occipital lobes, the pulvinar has attracted attention as a potential target for the treatment of TLE and PQE (95). In preoperative planning, direct targeting is commonly performed by integrating high-resolution imaging sequences, such as MPRAGE, with anatomical atlases.

Studies using stereoelectroencephalography (SEEG) suggest that the medial pulvinar may play a role in the propagation, amplification, and even termination of temporal lobe seizures (96, 97). It has also been implicated in impaired consciousness during seizures (98). Indeed, there are reports demonstrating improvement of temporal lobe seizures following direct stimulation of the pulvinar using SEEG electrodes (99).

Overall, the pulvinar appears to be particularly suitable for patients with TLE or PQE characterized by widespread epileptogenic networks. In addition, its relatively large size facilitates electrode placement, making it a favorable target for advanced neuromodulation strategies such as adaptive DBS and RNS.

### 3.4 Mediodorsal nucleus (DM)

The DM has rarely been used as a primary DBS target in clinical practice; however, its connectivity has attracted attention as a potential novel target for epilepsy treatment. The DM is located in the medial third of the thalamus, between the internal medullary lamina and the periventricular gray matter (100). Due to its anatomical position, it can be readily accessed during transventricular ANT-DBS procedures by placing the distal electrode contacts within the DM.

In a rat model of limbic seizures, stimulation of the DM resulted in greater than 80% seizure suppression (101). In contrast, other studies using amygdala kindling models have reported no significant antiseizure effects of DM stimulation (102). In a study of eight patients with drug-resistant focal epilepsy who underwent ANT-DBS, interictal activity recorded from both the ANT and DM revealed that some discharges were observed exclusively in the ANT, while others were detected only in the DM (103). These findings suggest the existence of epileptogenic networks involving the DM, supporting consideration of this region as a therapeutic target. Furthermore, in a case series examining multitarget DBS for DRE, seizure suppression was observed in patients receiving DM stimulation.

The DM can be subdivided into four subregions, which exhibit strong connectivity with the prefrontal cortex, orbitofrontal cortex, and anterior cingulate cortex (104). Through its involvement in frontolimbic networks, the DM is thought to modulate higher-order cognitive functions such as attention and working memory (105, 106). Consistent with this, therapeutic high-frequency stimulation of electrodes located in the DM during ANT-DBS has been reported to selectively impair working memory (107).

Taken together, DM-DBS may be particularly effective in epilepsy involving frontolimbic networks that include the DM (108).

### 3.5 Subthalamic nucleus (STN)

The evidence supporting the use of DBS targeting the STN in epilepsy is extremely limited, consisting mainly of case reports and small case series; however, therapeutic effects have been reported in several cases (109).

Historically, STN-DBS has been considered for DRE with seizure foci located in the central (sensorimotor) area, where resection is often not feasible. In a case series study, seizure reduction rates of 67–80% were observed in three out of five patients (110). In DRE patients with frontoparietal foci, a mean seizure reduction of 64% was observed with a minimum follow-up of 36 months (111). Treatment-related adverse events, including upper limb dyskinesia and dizziness, were reported in some cases but were manageable through reduction of stimulation intensity. The efficacy of STN-DBS has also been discussed in myoclonic seizures. Shan et al. (112) reported a case of refractory juvenile myoclonic epilepsy treated with STN-DBS, achieving an 87.5% reduction in seizure frequency at 1-year follow-up. Wille et al. (113) reported that combined substantia nigra pars reticulata (SNr)/STN-DBS resulted in seizure reduction ranging from 33 to 100% in five patients with progressive myoclonic epilepsy over a follow-up period of 12–42 months.

The mechanisms underlying seizure suppression by STN stimulation remain hypothetical. One proposed mechanism is that high-frequency stimulation directly modulates the cortico-subthalamic circuit, as well as the STN–SNr circuit, leading to activation of the dorsal midbrain anticonvulsant zone located in the superior colliculus, thereby exerting antiepileptic effects (109, 114, 115). Based on this hypothesis, STN-DBS may be particularly effective for focal epilepsy involving the sensorimotor cortex and for myoclonic seizures.

In addition, computational modeling studies have suggested that inhibitory projections from the STN to the cerebral cortex may contribute to the regulation of spike-and-wave discharges observed in absence seizures (116). Therefore, STN-DBS may also represent a potential therapeutic option for drug-resistant absence epilepsy.

### 3.6 Other targets

#### 3.6.1 Hippocampus

DBS targeting the hippocampus, a key structure in seizure onset, has been investigated for its antiseizure effects (117–119). In a prospective double-blind randomized controlled trial published in 2017 involving 16 patients with drug-resistant TLE, seven of eight patients in the stimulation group experienced a ≥ 50% reduction in seizure frequency during the 6-month blinded phase, and four patients became seizure-free (120). In an observational study of 25 patients with TLE who underwent hippocampal DBS (Hip-DBS) (10 unilateral and 15 bilateral cases), the median reduction in focal aware seizures was 66%, with seizure freedom achieved in 21% of patients (121). For focal impaired awareness seizures (FIAS), the median reduction was 91%, with a seizure freedom rate of 32%, indicating particularly strong efficacy for FIAS (121).

Choo et al. reported a retrospective observational study of bilateral Hip-DBS in 11 patients with bilateral TLE and four patients with occipital lobe epilepsy (122). After more than 1 year of follow-up, seizure reductions of 77.8% in the bilateral TLE group and 68.8% in the posterior epilepsy group were observed. In six patients who underwent pre- and postoperative neuropsychological assessments, no cognitive deterioration attributable to Hip-DBS was detected; moreover, although not statistically significant, improvements were observed in non-verbal reasoning, attention to detail, and visuospatial processing. Hip-DBS may therefore represent a valuable treatment option in cases where the hippocampus is clearly identified as the seizure focus, particularly in TLE involving the dominant hemisphere, where resection is less desirable due to the risk of functional deficits.

#### 3.6.2 Posteromedial hypothalamus

In DBS targeting the posteromedial hypothalamus for intractable aggressive behavior, a follow-up study of five patients with coexisting DRE over a period of up to 4 years demonstrated not only suppression of aggressive behavior but also a mean seizure reduction of 89.6% (123). Similar findings have been reported in other case studies, suggesting that modulation of limbic networks, including emotional and autonomic components, may secondarily contribute to seizure reduction (124).

#### 3.6.3 Nucleus accumbens

The nucleus accumbens, a target also investigated in major depressive disorder, has likewise been suggested as a potential target for epilepsy. In a study of five patients with DRE who underwent bilateral nucleus accumbens DBS, the median seizure reduction was 37.5% (125). Experimental studies in mouse models of TLE have shown that the nucleus accumbens modulates seizure propagation, which may underlie its potential antiseizure effects (126). These targets may represent promising options for patients with DRE accompanied by significant psychiatric comorbidities.

#### 3.6.4 Cerebellum

Attempts to apply DBS to the cerebellum in patients with DRE were first reported by Cooper et al. (5). While cerebellar stimulation has been proposed as a potential treatment for generalized tonic–clonic seizures, subsequent studies have yielded inconsistent results, and its efficacy remains unclear at present (127).

## 4 DBS for refractory status epilepticus (SE)

Status epilepticus (SE) is often associated with provoked seizures or acute symptomatic seizures, and therefore does not necessarily meet the criteria for epilepsy or DRE. However, this chapter focuses on DBS treatment for SE because it is an important neurological emergency associated with poor outcomes. Although the available evidence is limited to a small number of case reports, surgical intervention may be considered for the treatment of super-refractory status epilepticus (SRSE). Among these approaches, although the number of reported cases remains extremely limited, several patients have been treated with DBS. A PubMed search using the terms “status epilepticus” and “deep brain stimulation” identified 10 reported cases up to April 2026 (Table 2) (128–136). The patient age was 22.2 ± 17.4 years (mean ± SD, range 5–66 years), and five patients were male. The interval from the onset of SRSE to DBS implantation was 47.4 ± 18.4 days (range 27–80 days). The stimulation targets were CM in six patients, ANT in three patients, and the pulvinar in one patient. Following DBS initiation, all patients recovered from SRSE; however, persistent seizures and cognitive impairment were reported, and two patients remained in a vegetative state.

**Table 2:** Summary of emergency DBS cases for super-refractory status epilepticus.

Age/sex	Etiology	SE duration (pre-op)	DBS target	Improvement timeline	Outcome/prognosis	References
27/M	Unknown encephalopathy (post-cardiac arrest)	5 weeks	CM	Immediate disappearance of GTCS and GPEDs	Remained in vegetative state; died after lead removal due to infection 6 months later.	Valentin et al. (135)
17/M	Common variable immunodeficiency-associated encephalomyelitis	55 days	CM	10 days after starting stimulation (arousal reaction)	Returned home after 9 months of treatment/rehab; memory and executive deficits persist.	Lehtimäki et al. (131)
17/F	Absence status (Juvenile Absence Epilepsy)	4 weeks	ANT	Significant improvement within 1 week (arousal started on Day 2)	Discharged after 1 month; able to walk and speak; 90% reduction in seizures.	Lee et al. (130)
66/F	Unknown encephalopathy (cortical/subcortical)	30 days	ANT	Within 24 h after lead contact change	Dramatic cognitive recovery; seizure-free for 18 months.	Imbach et al. (129)
9/M	FIRES	Implanted on day 27	CM	Immediate cessation of generalized seizures (later combined with anakinra)	Discharged; returned to mainstream school; only short focal seizures remain.	Sa et al. (132)
5/M	FIRES	Implanted on day 37	CM	Generalized seizures stopped for 4 days immediately post-implant	Persistent vegetative state; focal seizures continued.	Sa et al. (132)
25/F	History of viral encephalitis (onset during pregnancy)	Approx. 2 months	ANT	SE resolved after stimulation adjustment (approx. Day 17 Post-op)	Well-managed for 2 years; died from SE recurrence after lead removal due to involuntary movements.	Yuan et al. (136)
15/F	Unknown (suspected focal cortical dysplasia)	80 days	CM	4 days after switching to low-frequency stimulation	SE resolved; subsequently underwent focal resection with 90% reduction in clinical seizures.	Stavropoulos et al. (133)
11/F	FIRES	56 days	CM	Arousal and alertness improved approx. 30 days after starting stimulation	Returned home after 4 months of rehab; short focal seizures remain.	Hect et al. (128)
30/M	Cryptogenic new onset refractory status epilepticus	66 days	Pulvinar	Seizures ceased on Day 8 Post-op	Alert and interactive but cognitive/functional status remained below baseline	Tang et al. (134)

DBS: deep brain stimulation, M: male, F: female, Pre-ope: before operation, Post-op: after operation, ANT: anterior nucleus of the thalamus, CM: centromedian nucleus, FIRES: febrile infection-related epilepsy syndrome, GTCS: generalized tonic–clonic seizure, GPEDs: generalized periodic epileptiform discharges, SE: status epilepticus.

One proposed mechanism by which DBS terminates status epilepticus is the modulation of specific neural nuclei, thereby preventing abnormal synchronization and propagation of epileptic discharges (129, 136). In addition to direct electrical effects, anti-inflammatory and anti-apoptotic actions, as well as modulation of cytokine balance, may also contribute (66, 67).

Overall, DBS may suppress otherwise pharmacoresistant SE by rebalancing the broader epileptic network and restoring the equilibrium between excitation and inhibition.

## 5 Current status and challenges

Although DBS represents an attractive therapeutic option for DRE, currently there are several challenges to be addressed. One major problem is the insufficient quality and quantity of clinical evidences, largely attributable to the limited number of reported cases. In particular, for targets other than the ANT, available data are largely restricted to small case series and retrospective studies. In addition, many aspects of long-term outcomes remain unclear, and continued follow-up may reveal changes in antiseizure efficacy as well as the emergence of previously unrecognized adverse events. Another important issue is that stimulation parameters have not yet been fully optimized. Therefore, further accumulation of cases and prospective studies are warranted, in parallel to identify the most effective stimulation setting according to characteristics of each patient.

## 6 Conclusion

DBS for epilepsy remains a palliative surgical treatment, and achieving complete seizure remission is often challenging. Nevertheless, it provides an important therapeutic option for patients who are not candidates for resective surgery or who continue to experience seizures after surgical intervention.

Appropriate selection among the various DBS targets requires a thorough understanding of the seizure types and epilepsy syndromes for which each target is most effective. This, in turn, necessitates a comprehensive understanding of epileptogenic networks, as well as detailed anatomical and physiological knowledge of each target. Accurate characterization of seizure semiology may help identify the neural networks involved, thereby facilitating the selection of more appropriate DBS targets. Careful evaluation of the underlying pathophysiology in individual cases is essential to determine the most effective therapeutic strategy.

Hitherto, many of these approaches have not yet been widely implemented in routine clinical practice. As these modalities of treatment become more broadly adopted, the range of therapeutic options available to epilepsy specialists is expected to expand. Further research is required to optimize each of these treatment strategies and to refine their clinical application.
